# Retraction: Identification of hub genes and construction of prognostic nomogram for patients with Wilms tumors

**DOI:** 10.3389/fonc.2023.1301668

**Published:** 2023-10-26

**Authors:** Frontiers Editorial Office

The journal retracts the 21 October 2022 article cited above.

Following publication, concerns were raised regarding the use of the SK-NEP-1 cell line in the study to investigate Wilms’ tumor. An investigation, which was conducted in accordance with Frontiers’ policies, confirmed that the SK-NEP-1 cell line has been reclassified as belonging to the Ewing sarcoma family of tumors, rather than of Wilm’s tumor origin.

It was found that the complaint was valid, and that the scientific validity of the article cannot be confirmed; therefore, the article has been retracted.

This retraction was approved by the Chief Editor of Frontiers in Oncology - Pediatric Oncology and the Chief Executive Editor of Frontiers. We thank the reader for raising this concern.

